# SMAC, a computational system to link literature, biomedical and expression data

**DOI:** 10.1038/s41598-019-47046-2

**Published:** 2019-07-19

**Authors:** Stefano Pirrò, Emanuela Gadaleta, Andrea Galgani, Vittorio Colizzi, Claude Chelala

**Affiliations:** 1 Bioinformatics Unit, Centre for Molecular Oncology, Barts Cancer Institute, Queen Mary University London, London, EC1M 6BQ UK; 2 Department of Biology, University of Rome Tor Vergata, Rome, Italy; 3 Interdepartmental Centre for Animal Technology, University of Rome Tor Vergata, Rome, Italy

**Keywords:** Literature mining, Data mining

## Abstract

High-throughput technologies have produced a large amount of experimental and biomedical data creating an urgent need for comprehensive and automated mining approaches. To meet this need, we developed SMAC (SMart Automatic Classification method): a tool to extract, prioritise, integrate and analyse biomedical and molecular data according to user-defined terms. The robust ranking step performed on Medical Subject Headings (MeSH) ensures that papers are prioritised based on specific user requirements. SMAC then retrieves any related molecular data from the Gene Expression Omnibus and performs a wide range of bioinformatics analyses to extract biological insights. These features make SMAC a robust tool to explore the literature around any biomedical topic. SMAC can easily be customised/expanded and is distributed as a Docker container (https://hub.docker.com/r/hfx320/smac) ready-to-use on Windows, Mac and Linux OS. SMAC’s functionalities have already been adapted and integrated into the Breast Cancer Now Tissue Bank bioinformatics platform and the Pancreatic Expression Database.

## Introduction

The NCBI PubMed^1^ is a biomedical literature-based search engine that provides data from MEDLINE®, life science journals and online books. It is the largest and most widely used resource for biomedical and scientific research, with over 27 million citations for biomedical literature available currently for querying.

In order to index the large amount of stored data, the National Library of Medicine (NLM) created a controlled vocabulary thesaurus named MeSH (Medical Subject Headings)^2^. MeSH descriptors are assigned to 16 categories, with each category divided into subcategories. In each subcategory, descriptors are arrayed hierarchically from most general to most specific in up to twelve hierarchical levels. Because of the branching structure of the hierarchies, these lists are sometimes referred to as “trees”. Each MeSH descriptor appears in at least one location in the tree, but it may appear in additional places if appropriate. Articles in PubMed are classified using multiple MeSH terms, from roots to leaves.

While PubMed offers simple and fast search capabilities, it is a daunting, not to mention time-consuming, task to wade through the sea of information retrieved^3^. For this reason, fast automatic extraction and integration of biological insights from biomedical literature represents a very attractive prospect.

Several automatic literature solutions were designed to identify, retrieve and extract information from a body of works based on user-defined search parameters. GoPubMed^4^ links PubMed articles with the Gene Ontology^5^ by parsing and categorising the abstracts. More recently, Frisch and colleagues developed LitInspector, a tool to provide gene and signal transduction pathway mining within PubMed^3^. Despite the first version being free of use, the resource is now part of the Genomatix® Software Suite and requires a license. PolySearch2^6^ is a text-mining approach that extracts associative relationships between biomedical entities, such as genes, proteins, human diseases, drugs, metabolites etc. A smart and user-friendly interface allows the user to conduct more than 60 unique combinations for each search. Similar to PolySearch2, pubmed.mineR^7^ combines the advantages of the existing algorithms with the flexibility provided by an R package.

Although very valuable, the aforementioned tools do not provide any kind of linkage or integration with the molecular data generated from the published studies. For these reasons we developed SMAC, a fast and automated method for collecting, prioritising, integrating and analysing biomedical data extracted from PubMed and Gene Expression Omnibus (GEO)^8^. The open-source nature of SMAC allows for add-on modules to be incorporated into the architecture thereby expanding the scope of the original software. SMAC is distributed as a docker container (https://hub.docker.com/r/hfx320/smac) and can be used out-of-box in any Windows, Mac or Linux system. The source code of SMAC is also available on GitHub (https://github.com/wynstep/SMAC).

Since its inception, SMAC has been employed successfully as a valuable module in Breast Cancer Now Tissue Bank bioinformatics^9^ and the Pancreatic Expression database^10^.

## Methods

SMAC is designed to extrapolate and link literature, biomedical and molecular data from user-defined queries and conduct bioinformatics analysis. It exploits latest NCBI programmatic access APIs and R packages to support either simple and complex queries, produced with a human-readable and -writeable syntax.

**Figure 1:**
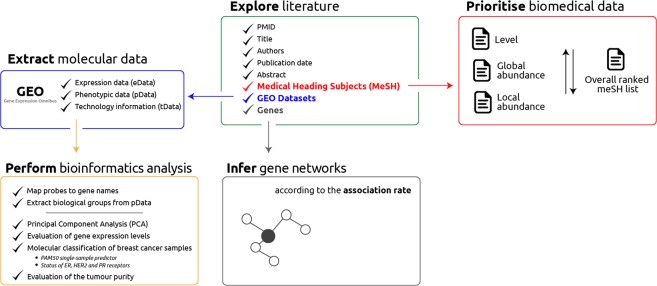
Schematic representation of the workflow performed by SMAC.

SMAC performs five main tasks during its execution (Fig. 1): (i) explore the literature by listing the most relevant manuscripts, according to the query; (ii) prioritise literature-related, biomedical data; (iii) create gene networks whereas strength and reliability of interactions is proportional to co-citation rate; (iv) extract expression data from GEO and convert it to a standard format; (v) perform specific bioinformatics analyses, based on user selections.

### Starting the analysis

SMAC is able to support two main operative situations. Users can either extract all relevant publications and their associated data starting from a text-free user-defined query or use SMAC to retrieve information, and subsequent data, from a defined list of PubMed IDs (PMIDs). Text-free search terms, defining the concepts of interest (topic or list of PMIDs), and an email address must be defined to launch the data selection and retrieval process. To speed up an analysis, users can limit the amount of results retrieved and/or bypass the recovery of expression data.

### Retrieval and prioritisation of literature data

SMAC exploits the Entrez Programming Utilities to mine biomedical literature and identify the most relevant articles. Records are then ranked according to the “Best Match” relevance algorithm^11^ that takes into consideration different factors i.e. past usage of an article, publication date, number of citations etc. For each publication reclaimed, a comprehensive set of information is collected (Table 1).

**Table 1:** Description of the metadata retrieved for each publication.

Type of information	Description
PMID	Unique identifier number for publications stored in PubMed
Title	Full title of the publication
Authors	List of authors, separated by comma
Journal	Full name of the journal that published the paper
Date of publication	Date of publication is always reported using the *yyyy-mm-dd* format
MeSH headings	List of the medical headings associated to the publication
GSE codes	List of the GEO dataset linked to the PMID
Platforms	Experimental platforms used for generating the data
ftp-links	Web link for the direct download of the GEO raw data
Analyses	List of analyses performed by SMAC on each tuple *PMID:GSE*

### Prioritisation of biomedical data

Medical Subject Headings (MeSH) represent a reliable bulk of terms for connecting the literature and the biomedical layers. Among the subjects retrieved, some are more important than others. For this reason, it’s crucial to apply a prioritisation procedure that takes into consideration the (i) hierarchical level (specificity), (ii) abundance in topics-related articles and (iii) abundance in all PubMed citations. The prioritisation workflow is composed of two main parts: first, MeSH terms are sorted separately according to each criteria, second the Robust Ranking Aggregation method^12^ prioritises the most statistically-relevant elements by detecting those that are ranked consistently better than expected under the null hypothesis of the random allocation of items.

### Retrieval and manipulation of expression data

The integration of molecular data generated from published studies supersedes the functionality of all the existing tools. SMAC takes advantage of the R/Bioconductor package *GEOquery*^13^ to retrieve expression datasets from NCBI Gene Expression Omnibus^8^. For each GEO series (GSE), three data packages are generated in order to reflect sample-level granularity:

*pData* includes the phenotypic and experimental information deposited by the research group. SMAC applies a text-mining approach to stratify samples into different biological groups. Moreover, cancer samples are identified and separated from normal/controls. This step is crucial for performing a subset of analyses, particularly designed for tumour data.

*eData* packs the expression levels belonging to each sample.

*tData* reports the information related to the technology used for generating the data, as well as a conversion dictionary between probes and gene names. The presence of *tData* is fundamental to reduce the dimensionality of *eData* as it allows for the merging expression levels belonging to the same gene, thereby facilitating subsequent bioinformatics analyses.

### Bioinformatics analysis

SMAC incorporates a body of bioinformatics analysis to be applied on the *eData* extracted and manipulated from GEO. While the core analyses can be applied to any kind of expression data, regardless of the biological context, a subset of analyses are cancer-specific and can only be applied to cancer-related datasets. Results are provided by SMAC in a shape of interactive graphs generated by the *plotly* R package (https://plot.ly).

#### Principal Component Analysis (PCA) – Core analysis

**Figure 2:**
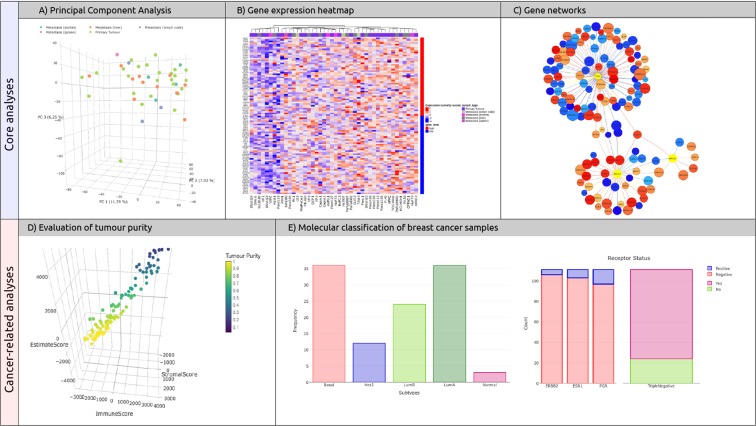
Bioinformatics analyses performed by SMAC. The Principal Component Analysis **(A)** permits to highlight the key sources of variation. Gene expression heatmap **(B)** shows the normalised levels for the most variable genes. An interactive gene network **(C)** reflects the association rate among the genes in selected publications. The cellular purity of cancer samples is presented in a single, interactive scatterplot **(D)**. Two interactive barplots **(E)** show the percentage of breast cancer samples belonging to each molecular subtype and receptor status profile.

A PCA reduces the complexity of multidimensional data while minimising the loss of information and preserving data structure^14^. A set of “components” are extracted from the expression dataset, by linearly combining the original genes. Data are transformed into a coordinate system and presented as an orthogonal projection. The outcome of this analysis is reported by SMAC in a form of a 2D/3D scatterplot where the position of samples, reflects their mutual similarity (Fig. 2A). As an explorative analysis, PCA captures the presence of clusters of samples showing similar expression patterns.

#### Gene expression levels – Core analysis

The normalised expression levels (z-scores) for the most variable genes (n = 20, n = 50, n = 100 or an arbitrary number decided by the user) is presented across all samples in the GEO dataset. Moreover, samples are clustered according to their expression profiles for the subset of genes. This analysis produces a heatmap where rows and columns represent genes and samples, respectively (Fig. 2B).

#### Gene interaction network – Core analysis

Using the set of papers stored in the literature layer, SMAC uses the Entrez Programming Utilities *elink* to extract the genes correlated to the publications, together with a set of scores that reflects their association rate. SMAC then implements the R package *visNetwork* (http://datastorm-open.github.io/visNetwork/) to produce an interaction network by overlapping the genes with the, manually-curated, Mentha interactome^15^. Nodes (genes) are coloured according to their association-score while edges (connection between genes) are weighted according to the Mentha scoring system (Fig. 2C).

#### Tumour purity – Cancer related

The cellular purity of cancer samples is often affected by the presence of small amounts of infiltrating stromal and immune cells that may confound subsequent analyses. If SMAC detects the presence of cancer samples, it will apply the ESTIMATE algorithm^16^ to infer the tumour purity from the corresponding expression data. This results in an interactive 3D scatterplot that correlates all the calculated scores (Stromal, Immune and ESTIMATE), where samples (dots) are coloured according to their purity percentage (Fig. 2D).

#### Molecular classification – Cancer related

Molecular classification models are applied to datasets comprising breast cancer samples. First, the PAM50 single sample predictor, is used to predict the molecular subtype of each sample —Luminal A (LumA), Luminal B (LumB), Basal-like (Basal), Her2-enriched (Her2) and Normal breast-like (Norm). Next, the molecular status of key breast cancer receptors, oestrogen, progesterone and Her2, is estimated using *mclust*. Results are presented as interactive bar plots showing the percentage of samples belonging to each molecular subtype and receptor status profile (Fig. 2E).

### Distribution of the software

SMAC has been developed using Python and R, and is distributed to public in a form of Docker package (https://hub.docker.com/r/hfx320/smac). Thanks to its modularity, users can easily edit the source code of SMAC (available on GitHub at https://github.com/wynstep/SMAC) by implementing R-based, custom analyses to be included in the main pipeline. Further analytical modules will be also implemented in future releases of the software.

## Results

### Semantic similarity with Polysearch2 database

To evaluate the reliability of the terms retrieved by SMAC, we conducted three biomedical tests focussed on diabetes, multiple sclerosis and metformin. The *meshes* R package^17^ was used to calculate the semantic similarity among SMAC and Polysearch2 terms (adopted as Gold Standard). A wide range of semantic, similarity measures were explored: Shortest-Path^18^, Weighted-Link^19^, Wu and Palmer^20^, Leacock and Chodorow^21^, Li^22^ and Lord^23^.

Apart from Lord’s metrics, all the others are defined as path-based similarity measures and assume that the hierarchy of headings is organised along the lines of semantic similarity. Lord’s metrics is an Information-based value and is correlated with the frequency of the heading in a given document collection. All the scores are normalised between 0 and 1 and represent the probability of two sets of MeSH terms to be similar. For this reason, a value of 0.5 (50% probability) is often used as minimum threshold to select the statistically significant comparisons^24^.

**Figure 3:**
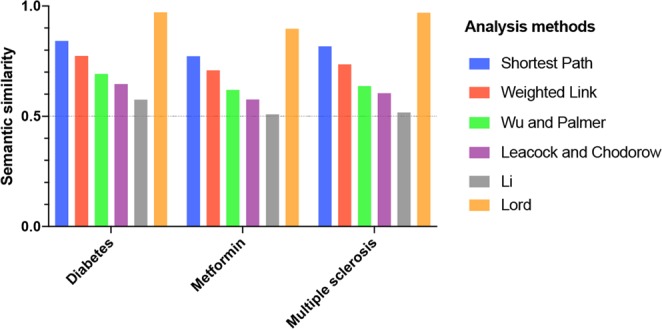
Semantic similarity benchmarks between SMAC and Polysearch2. The value of 0.5 is set as minimum threshold for statistically significant comparisons.

The benchmark conducted against Polysearch2 shows that all the path-based similarity measures have a value higher than 0.5, with the Shortest-Path methods achieving peaks of 0.84 when comparing Diabetes-related terms (Fig. 3). Lord’s metric shows similarity measures between 0.89 and 0.97 in all the tests, demonstrating that SMAC is able to capture the biomedical insights correctly.

In our comparison benchmarks, path-based tests can be considered more stringent. In fact, two MeSH terms will be considered more similar if they share the same hierarchical level (specificity) and the semantics. On the other hand, Lord’s method takes into account both the semantic similarity and the frequency of the term in the PubMed dataset.

### Comparison with other tools

SMAC represents a cutting-edge technology in terms of data mining. To the best of our knowledge, no other method offers users the ability to link and integrate the literature and biomedical information in PubMed with the -omics data stored in GEO. Table 2 provides a comparison of SMAC with other tools that have been developed for re-analysing datasets from GEO including GEO2R^8^, shinyGEO^25^, GEOquery^13^, ImaGEO^26^, ScanGEO^27^, GEO2Enrichr^28^ and BART^29^. SMAC is the only tool that has been designed and developed to run locally, all results are retrieved on-the-go from the NCBI servers, then downloaded and analysed on the host machine. There is a potential to include enrichment and meta analyses modules in the next release of SMAC.

**Table 2:** Comparison of SMAC with other tools focused on the reanalysis of GEO datasets.

Tool	Description	Single/multiple	Type of analyses					
			PCA	DEGs	Tumour purity	Molecular classification	Enrichment Analysis	Meta-analysis
SMAC	Download and analyse multiple GEO datasets	Multiple	✓	✓	✓	✓	✗	✗
GEO2R	Compares two or more groups of samples in a GEO dataset	Single	✗	✓	✗	✗	✗	✗
shinyGEO	Shiny extension of GEO2R	Single	✗	✓	✗	✗	✗	✗
GEOquery	R package for **downloading** GEO datasets	Single	✗	✗	✗	✗	✗	✗
ImaGEO	Meta-analyses across multiple GEO studies	Multiple	✗	✗	✗	✗	✗	✓
ScanGEO	Identifies Differentially Expressed Genes across multiple GEO studies	Multiple	✗	✓	✗	✗	✗	✗
GEO2Enrichr	Performs enrichment analyses on DEGs extracted from GEO datasets	Single	✗	✓	✗	✗	✓	✗
BART	Download and analyse microarray data from GEO	Multiple	✓	✗	✗	✗	✓	✗

### Evaluation of the computational speed

**Figure 4:**
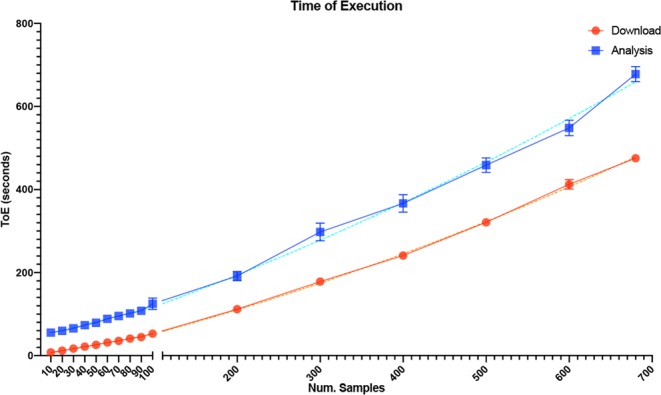
Evaluation of the computational burden for the download and analysis tasks. Both curves follow a polynomial, quadratic trend, represented as dashed lines.

We evaluated the computational speed of SMAC by calculating the Time of Execution (ToE) for downloading and analysing an increasing number of invasive breast cancer samples from GEO (GSE102484^30^). A  local machine with 2 Xeon 5600 processors and 6GB of RAM was used. All the analysis currently implemented in SMAC have been applied on each downloaded dataset. As reported in Fig. 4 there is a positive correlation between the ToE and the number of samples, for both downloading and analysing the data and an overall speed of less than 1 s per sample.

### Adaption of SMAC by BCNTB bioinformatics and the Pancreatic Expression database

SMAC has been integrated successfully into the infrastructure of BCNTB bioinformatics and PED. This model was employed to reduce the burden and time required to select and curate the data manually. These cancer initiatives have expanded the functionality of SMAC by incorporating multiple analytical modalities into its base code. The adoption and expansion of SMAC by BCNTB bioinformatics and PED has allowed for an exponential growth in the data available to the breast and pancreatic cancer research community.

SMAC cross-references entries in PubMed with cancer-specific domains (controlled vocabulary terms). For each entry identified by SMAC, the PubMed identifier, title, authors, publication date and abstract are extracted and made available to researchers.

BCNTB bioinformatics and PED comprise both data mining and analytical components. For the latter, a secondary identification stage was incorporated into SMAC to replace a second manual curation step. Attributes relating to the submission of experimental data, such as GEO identifiers, are extracted and computational links between the entry and its associated experimental data established. If data is publicly available, SMAC accesses and downloads the relevant data files. These are fed into the analytical pipelines developed for each resource automatically.

Adoption of SMAC has allowed for automation of the data retrieval, extraction, preparation and analysis process. Furthermore, this system opens up the opportunity for periodic enrichment of the resources with minimal manual intervention. These cancer resources are freely available from http://bioinformatics.breastcancertissuebank.org^9^ and http://www.pancreasexpression.org^10^.

## Conclusions

We designed SMAC, the Smart Automatic Classification system (https://hub.docker.com/r/hfx320/smac) to bridge literature information, biomedical headings and molecular data. Starting from a text-free, user-defined query, SMAC collects and prioritises all the topic-related publications in PubMed. A set of biomedical terms (MeSH) are also extracted and ranked according to multiple features (specificity, local and global abundances). Unlike other tools available, SMAC integrates and slims the molecular data generated from published studies. A set of core and, where relevant, cancer-specific bioinformatics analyses are applied on the retrieved datasets and outcomes are reported in an interactive fashion. A benchmark with Polysearch2 clearly highlights the reliability of SMAC to extract the biomedical insights from the literature layer.

The modularity of the architecture of SMAC permits custom modules to be incorporated, expanding its functionality. SMAC has been already adopted by two important cancer resources focused on breast and pancreatic cancer and, in future, aims to be incorporated into more biomedical resources.

## Data Availability

https://hub.docker.com/r/hfx320/smac.
